# Tumor Suppressor CYLD Acts as a Negative Regulator for Non-Typeable *Haemophilus influenza*-Induced Inflammation in the Middle Ear and Lung of Mice

**DOI:** 10.1371/journal.pone.0001032

**Published:** 2007-10-10

**Authors:** Jae Hyang Lim, Hirofumi Jono, Tomoaki Koga, Chang-Hoon Woo, Hajime Ishinaga, Patricia Bourne, Haodong Xu, Un-Hwan Ha, Haidong Xu, Jian-Dong Li

**Affiliations:** 1 Department of Microbiology and Immunology, University of Rochester Medical Center, Rochester, New York, United States of America; 2 Cardiovascular Research Institute, University of Rochester Medical Center, Rochester, New York, United States of America; 3 Department of Pathology and Laboratory Medicine, University of Rochester Medical Center, Rochester, New York, United States of America; Centre de Recherche Public-Santé, Luxembourg

## Abstract

Non-typeable *Haemophilus influenza* (NTHi) is an important human pathogen causing respiratory tract infections in both adults and children. NTHi infections are characterized by inflammation, which is mainly mediated by nuclear transcription factor kappaB (NF-κB)-dependent production of inflammatory mediators. The deubiquitinating enzyme cylindromatosis (CYLD), loss of which was originally reported to cause a benign human syndrome called cylindromatosis, has been identified as a key negative regulator for NF-κB *in vitro*. However, little is known about the role of CYLD in bacteria-induced inflammation *in vivo*. Here, we provided direct evidence for the negative role of CYLD in NTHi-induced inflammation of the mice *in vivo*. Our data demonstrated that CYLD is induced by NTHi in the middle ear and lung of mice. NTHi-induced CYLD, in turn, negatively regulates NTHi-induced NF-κB activation through deubiquitinating TRAF6 and 7 and down-regulates inflammation. Our data thus indicate that CYLD acts as a negative regulator for NF-κB-dependent inflammation *in vivo*, hence protecting the host against detrimental inflammatory response to NTHi infection.

## Introduction

Nontypeable *Haemophilus influenzae* (NTHi), a gram-negative bacterium, is an important human pathogen in both adults and children [1]. In adults, it exacerbates chronic obstructive pulmonary diseases, and in children, it causes otitis media, the most common childhood infection and the leading cause of conductive hearing loss [2]–[4]. Despite the need for prophylactic measures, development of a vaccine for preventing NTHi infections has been difficult and still remains a great challenge. Moreover, inappropriate antibiotic treatment contributes to the worldwide emergence of antibiotic-resistant strains of NTHi. Therefore, there is an urgent need for developing alternative therapeutic strategies for the treatment of NTHi infections based on understanding the molecular pathogenesis of NTHi infections. Like most other bacterial infections, NTHi infection is characterized by inflammation, which is mainly mediated by nuclear factor kappaB (NF-κB)-dependent production of pro-inflammatory mediators [5]–[8]. We have previously shown that NTHi induces Toll-like receptor (TLR) 2-dependent activation of NF-κB via an IKKβ-IκBα- and p38 mitogen-activated protein kinase (MAPK)-dependent signaling pathway [6], [9], [10]. However, the key signaling adaptors that link TLR2 with IKK and MAPK in mediating NTHi-induced inflammation remain unknown.

Although the inflammatory response triggered by bacteria is essential for eradicating bacterial pathogen, excessive inflammatory response is clearly detrimental to the host, due to severe tissue damage [11], [12]. To avoid overactive and detrimental inflammatory response in infectious disease, the bacteria-induced inflammatory response must be tightly regulated. During evolution, the host has developed a variety of strategies to prevent detrimental inflammatory response during bacterial infections. Among all strategies, bacteria-induced negative feedback regulation is thought to play a critical role in preventing overactive inflammatory response by tightly regulating the activity of the key receptor-dependent signaling adaptors specifically activated by bacterial pathogens. In the pathogenesis of chronic obstructive pulmonary diseases and otitis media, to avoid detrimental inflammatory responses in NTHi infection, TLR2 signaling must be tightly regulated. However, despite the importance of tight regulation in preventing overactive inflammatory response, the molecular mechanisms underlying the negative feedback regulation of inflammation in the pathogenesis of NTHi infection remain unknown.

CYLD was originally identified as a tumor suppressor, loss of which causes a benign human syndrome called cylindromatosis [13]–[15]. *In vitro* studies have indicated that CYLD is a member of the deubiquitinating enzyme family that specifically digests polyubiquitin chains. Transfection studies showed that CYLD deubiquitinates TRAF2 and TRAF6 and acts as a negative regulator for activation of NF-κB by tumor necrosis factor receptor (TNFR) and TLR [16]–[18]. Recently, we, together with others, showed that CYLD also negatively regulates activation of MAPKs, including p38 MAPK [19]–[22]. Moreover, the expression of CYLD is itself under the control of NF-κB [20], [23], suggesting that CYLD is involved in a negative feedback regulation of NF-κB activation and NF-κB-dependent gene expression. Given the important role that NF-κB plays in host immune and inflammatory response in bacteria infection, it is logical to hypothesize that CYLD may act as a negative regulator for immune and inflammatory response against invading bacteria such as NTHi *in vivo*. However, despite recent studies demonstrating the role of CYLD in regulating T cell receptor signaling and tumor cell proliferation *in vivo*, the biological role of CYLD especially its negative role in inflammation *in vivo* still remains unknown.

In the present study, by using NTHi-induced otitis media and pneumonia model in wild type (WT) and *Cyld*-deficient mice, we provide *in vivo* evidence that NTHi induces pro-inflammatory response through TLR2-dependent MyD88-TRAF6/7-NF-κB signaling pathway, and CYLD negatively regulates NF-κB-dependent inflammatory response by NTHi via deubiquitination of TRAF6 and TRAF7. These studies may lead to development of novel therapeutic strategies for controlling overactive inflammatory response in respiratory bacterial infections.

## Materials and Methods

### Mice

*Cyld^−/−^* mice were generated by homologous recombination as previously reported [21]. A *Cyld*-gene targeting construct was designed to delete exons 2 and 3 and replace them with a lacZ reporter and a neomycin resistance gene. The targeting vector was linearized and electroporated into 129S ES cells. Clones resistant to G417 were selected and screened for homologous recombinants by Southern blot analysis. Two targeted ES cell clones were microinjected into C57BL/6 blastocysts, and the resulting chimeras were mated to C57BL/6 females to generate mice heterozygous for the *Cyld* mutation. Homozygous knockout animals were obtained by mating of heterozygous males and females. Genotyping was performed by PCR on tail-derived genomic DNA, and absence of RNA transcript and protein expression was confirmed by RT-PCR and Western blot analysis. *Tlr2^−/−^* and *MyD88^+/−^* were described previously [24].

### Animal experiments

For NTHi-induced otitis media model in WT and *Cyld^−/−^* mice, anaesthetized mice were transtympanically inoculated with 1×10^7^ CFU of NTHi under the surgical microscope, and saline was inoculated as control. The inoculated mice were then sacrificed by intraperitoneal inoculation of 100 mg/kg sodium pentobarbital at 3, 6, 9, 24, 72, and 168 hours after inoculation of NTHi. Eardrum was inspected under the otoscope and findings were recorded. To assess the mRNA expression of inflammatory mediators, total RNA was extracted from the bulla of NTHi and saline-inoculated ear at the time points indicated above and real-time quantitative PCR (Q-PCR) was performed as described previously [20]. For histological analysis, dissected temporal bones were fixed with 10% buffered formaldehyde overnight with rocking, decalcified with CalEX, embedded in paraffin, and sectioned at 5-µM thickness. Sections were then stained with hematoxylin and eosin (H&E) to visualize inflammatory response and pathological changes in the middle ear. H&E-stained middle ear sections were then evaluated by light microscopy using Axiovert 40 CFL (Carl Zeiss), and images were recorded with an AxioCam MRC (Carl Zeiss). NTHi culture positivity was confirmed by bacterial culture of middle ear effusion from NTHi-inoculated ears.

For NTHi-induced pneumonia model in WT and *Cyld^−/−^, Tlr2^−/−^,* and *MyD88^+/−^* mice, anaesthetized mice were intratracheally inoculated with 5×10^7^ CFU of NTHi, and saline was inoculated as control. The inoculated mice were then sacrificed by intraperitoneal inoculation of 100 mg/kg sodium pentobarbital at the time points indicated above. For histological analysis, dissected lung was inflated and fixed with 10% buffered formaldehyde, embedded in paraffin, and sectioned at 5-µM thickness. Sections were then stained and inspected as described above. For polymorphonuclear neutrophil (PMN) analysis, bronchoalveolar lavage (BAL) was performed by cannulating the trachea with sterilized PBS. Cells from BAL fluid were stained with Hemacolor (EM Science) after cytocentrifugation (Thermo Electronic Co.). To assess the mRNA expression of inflammatory mediators, total RNA was extracted from the lung of NTHi and saline-inoculated lung at the time points indicated above and Q-PCR was performed as described previously [20]. All animal experiments were approved by the Institutional Animal Care and Use Committee at University of Rochester.

### Bacteria strain and culture

Clinical isolate of NTHi wild-type strain 12 was used in *in vitro* cell culture experiments and *in vivo* animal experiments [9]. Bacteria was grown on chocolate agar at 37°C in an atmosphere of 5% CO_2_ for overnight and inoculated in brain heart infusion broth supplemented with 3.5 µg of NAD per ml (BHI). After overnight incubation, bacteria were subcultured into 5 ml of fresh BHI and the log phase NTHi that was monitored by measurement of optical density (OD) value was washed and suspended in phosphate-buffered saline for *in vitro* cell experiments and in isotonic saline for *in vivo* animal experiments. For *in vitro* experiments, the epithelial cells were treated with NTHi at a multiplicity of infection (MOI) of 1:25 for various times as indicated. For *in vivo* animal experiments, NTHi was inoculated into the middle ear for otitis media model and the lung for pneumonia at a concentration of 1×10^7^ CFU/ear and 5×10^7^ CFU/lung, respectively.

### Cell culture

Human middle ear epithelial cell line HMEEC-1, a commonly used middle ear cell line, was derived by human papilloma virus immortalization of primary human middle ear epithelial cells, and was maintained in a 1∶1 mixutre of Bronchial Epithelial Basal Medium (BEBM) and Dulbecco's modified Eagle's medium (DMEM) as described [6], [9], [25]. Airway epithelial cell line A549 was maintained as described [9], [26]. Primary normal human bronchial epithelial cell (NHBE) were purchased from Cambrex and were maintained as described previously in Bronchial Epithelial cell Growth Medium (BEGM) [6], [23], [25]. For air-liquid interface culture, NHBE cells were cultured as described previously [27]. In brief, NHBE cells were seeded at 2×10^4^ cells/cm^2^ onto 24-mm-diameter, 0.4 µm pore size, semi-permeable membrane inserts (Corning) in BEBM. The cultures were grown submerged for the first 7 days and then, the air-liquid interface was created by removing media from the apical compartment of the cultures. The culture media were changed every other day until the air-liquid interface was created and were changed daily by replacing fresh media only to the basal compartment during the air-liquid interface culture. NHBE cells were grown in air-liquid interface for 2–3 weeks before being used for the proposed experiments. All cells were cultured in a humidified atmosphere of 5% CO_2_ and 95% air at 37°C.

### Transfection and Small Interfering RNA (siRNA)

The plasmids TLR2, MyD88, TRAF6, and TRAF7 DN, WT CYLD, and NF-κB-luciferase reporter were described previously [6], [9], [20], [23], [25]. Cells were cultured on 24-well plates. After 24 hours, cells were co-transfected with or without NF-κB-luciferase reporter plasmid and various expression plasmids as indicated in the figure legends. Empty vector was used as a control and was added where necessary to ensure a constant amount of input DNA. All transient transfections were carried out in triplicate using a TransIT-LT1 reagent (Mirus Co.) following the manufacturer's instructions. At 40 hours after the start of transfection, cells were inoculated with NTHi for 5 hours before cell lysis for luciferase assay or mRNA analysis as described previously. RNA-mediated interference for down-regulating CYLD expression was done using siRNA-CYLD as described previously [20], [23].

### Western blot (WB) analysis and immunoprecipitation (IP)

Antibodies against TRAF6, HA and Ubiquitin were purchased from Santa Cruz Biotechnology, and CYLD was from IMGENEX.

Western blots were performed as described [20], [23] and following the manufacturer's instruction. Briefly, Western blots were performed using whole cell extracts, separated on 8–10% SDS-PAGE gels, and transferred to poly-vinylidine difluoride membranes. The membrane was blocked with 5% nonfat milk, incubated in a 1:2,000 dilution of a primary antibody, and incubated with 1∶2,000 dilution of the corresponding secondary antibody. The membrane was reacted with chemiluminescence reagent ECL to visualize to blots. For immunoprecipitation, cell lysates were immunoprecipitated with 1 µg of the appropriate antibodies for overnight at 4°C and then conjugated to protein A/G-agarose beads for 2 hours at 4°C.

### Statistical analysis

Data were analyzed using Student's t-test. A value of *p<0.05* was considered significant.

## Results

### *Cyld^−/−^* mice are hyperresponsive to NTHi-induced inflammation in the ear and lung of the mice

To determine the biological role of CYLD in regulating inflammation *in vivo*, *Cyld*-deficient mice were generated by using standard targeting technique as described previously [21]. Briefly, *Cyld^−/−^* mice were generated by replacing exons 2 and 3 of *Cyld* gene with a neomycin resistance-lacZ cassette. Homozygous mutant was ascertained by PCR genotyping from tail tissue of mutant mice. RT-PCR analysis confirmed the absence of *Cyld* transcripts in *Cyld^−/−^* mice, and immunoblot analysis of mouse embryonic fibroblast (MEF) using an antibody against CYLD showed *Cyld* deficiency. *Cyld*-deficient mice exhibit no overt abnormalities and have a normal lifespan.

**Figure 1:**
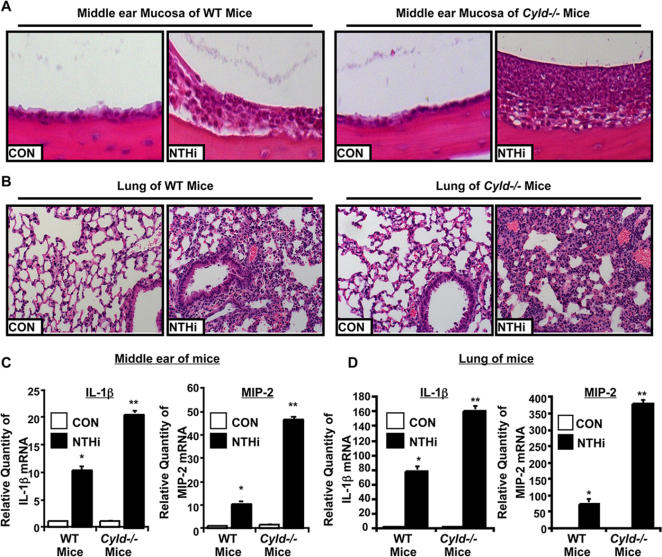
* Cyld^−/−^* mice are hyperresponsive to NTHi-induced inflammation in the ear and lung of the mice. A, NTHi was trans-tympanically inoculated into the middle ears of WT and *Cyld^−/−^* mice and bullaes were dissected from WT and *Cyld^−/−^* mice inoculated with NTHi and saline for the control for histological analysis (H&E stain, 200X). B, NTHi was intratracheally inoculated into the lungs of WT and *Cyld^−/−^* mice and lung tissues were dissected from WT and *Cyld^−/−^* mice inoculated with NTHi and saline for the control for histological analysis (H&E stain, 200×). C & D, NTHi was trans-tympanically (C) or intratracheally (D) inoculated into the middle ear (C) or lung (D), respectively, of WT and *Cyld^−/−^* mice and mRNA expression of the inflammatory mediators, IL-1β and MIP-2, was measured by Q-PCR analysis *p<0.005 compared with control inoculation in WT mice, **p<0.05 compared with NTHi inoculation in WT mice. Values are means ± S.D. (n = 3). *CON*, control.

We next investigated the negative role of CYLD in inflammatory response against NTHi infection in the middle ear and lung of *Cyld^−/−^* mice. Age- and sex-matched WT and *Cyld^−/−^* mice were inoculated with NTHi and inflammatory response in the middle ear and lung of infected mice was monitored up to 7 days. Histological examination of the middle ear revealed enhanced inflammatory responses including enhanced leukocyte infiltration in *Cyld^−/−^* mice compared with WT mice (**Fig. 1A**). Consistent with this result, histological evaluation of the lung of NTHi-inoculated mice showed enhanced leukocyte infiltration in peribroncheal and interstitial area in *Cyld^−/−^* mice compared with WT mice (**Fig. 1B**).

NTHi-induced inflammatory response and leukocyte infiltration are mediated by pro-inflammatory mediators, including IL-1β and MIP-2 [5], [6]. Thus we measured expression of IL-1β and MIP-2 at mRNA levels in the middle ear and lung from both WT and *Cyld^−/−^* mice 9 hours after NTHi inoculation. Compared with WT mice, *Cyld^−/−^* mice showed higher levels of IL-1β and MIP-2 mRNA expression in the middle ear (**Fig. 1C**) and in the lung (**Fig. 1D**). In addition, we also found significant increases in the levels of TNF-α mRNA expression in *Cyld^−/−^* mice (data not shown). These results demonstrate that CYLD plays an important role in negatively regulating NTHi-induced inflammatory response *in vivo*.

### NTHi-induced CYLD is responsible for down-regulation of inflammatory response

**Figure 2:**
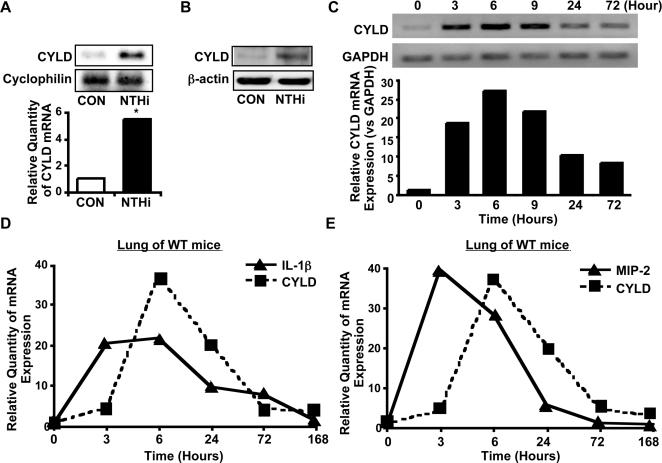
NTHi-induced CYLD is responsible for down-regulation of inflammatory response. A & B, NTHi induced CYLD expression at both mRNA (A) and protein levels (B) in HMEEC-1 cells. C, NTHi induced CYLD expression at the mRNA level in the middle ear of WT mice. D & E, NTHi induced expression of CYLD and IL-1β (D) or MIP-2 (E) at the mRNA level in the lung of WT mice. *p<0.05 compared with CON. Values are means ± S.D. (n = 3). *CON*, control.

It is well known that a variety of genes involved in inflammatory response undergo changes in expression pattern in response to bacterial infection [28]–[30]. We recently found that the endogenous expression level of CYLD is relatively low in middle ear and lung under physiological condition [13], but highly inducible by bacterial pathogens [20], [23], we hypothesized that CYLD is induced by NTHi and increased CYLD expression will in turn lead to inhibition of NTHi-induced inflammatory response, thereby preventing overactive inflammatory response that is detrimental to the host. We tested our hypothesis by first evaluating the effect of NTHi on CYLD expression. As shown in **Fig. 2A**, NTHi induced CYLD expression at the mRNA level in middle ear HMEEC-1 cells. The induction of CYLD by NTHi was also confirmed at the protein level by performing Western blot analysis (**Fig. 2B**). Similar result was also observed in human airway A549 cells and the primary human airway epithelial cells cultured under both regular liquid culture and physiological air-liquid interface condition (Data not shown). Moreover, up-regulation of CYLD by NTHi was also observed in the middle ear of NTHi-inoculated WT mice (**Fig. 2C**). Marked induction of CYLD by NTHi was observed at 6–9 hours, and an even greater induction of CYLD was still observed at 3–7 days. Consistent with these findings, marked induction of CYLD by NTHi was observed in the lungs of NTHi-inoculated WT mice along with down-regulation of pro-inflammatory mediators, IL-1β and MIP-2 (**Fig. 2D & E**). It is interesting to note that the peak of CYLD induction was clearly preceded by the peak of the induction of IL-1β and MIP-2, thereby suggesting that NTHi-induced CYLD is responsible for down-regulation of inflammatory response.

### NTHi induces inflammatory response through TLR2-MyD88-TRAF6/7-NF-κB signaling pathway

**Figure 3:**
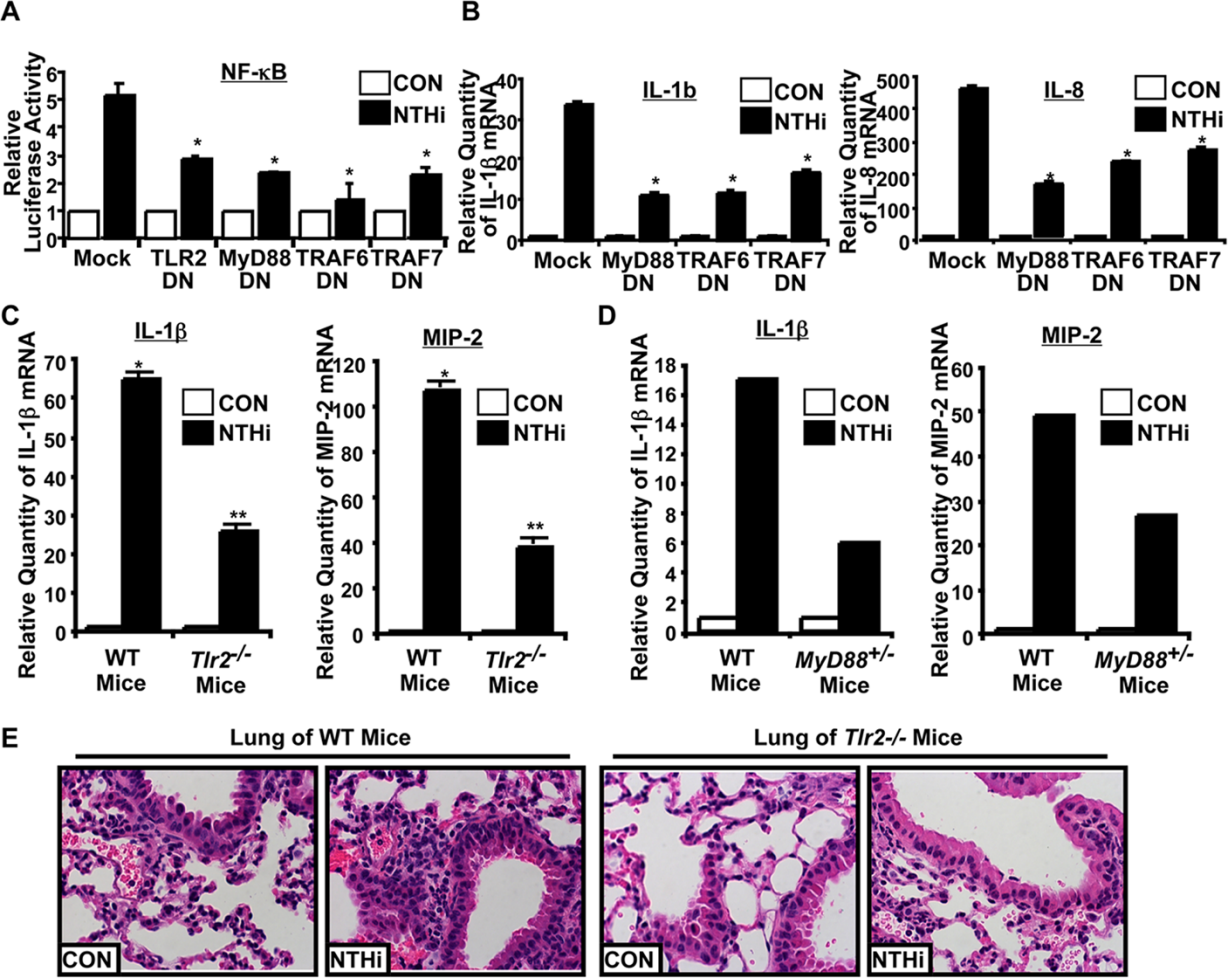
NTHi induces inflammatory response through TLR2-MyD88-TRAF6/7-NF-κB signaling pathway. A & B, Overexpressing dominant-negative (DN) mutants of TLR2, MyD88 and TRAF6/7 inhibited NTHi-induced activation of NF-κB (A) and up-regulation of IL-1β and IL-8 (B) in HMEEC-1 cells. *p<0.001, compared with Mock. Values are means ± S.D. (n = 3). C, NTHi-induced expressions of IL-1β and MIP-2 mRNA were reduced in the lung of *Tlr2^−/−^* mice *in vivo*. *p<0.005, compared with CON in WT mice, **p<0.05 compared with NTHi inoculation in WT mice. Values are means ± S.D. (n = 3). D, NTHi-induced expressions of IL-1β and MIP-2 were reduced in the lung of *MyD88^+/−^* mice *in vivo*. E, Histological analysis of lung of *Tlr2^−/−^* mice revealed reduced inflammatory response by NTHi compared with WT mice (H&E stain, ×200).

We next sought to identify the molecular target of CYLD in negatively regulating NTHi-induced inflammation. We first examined if TLR2-MyD88 and TRAFs, a family of adaptor proteins, are critically involved in mediating NTHi-induced inflammation. As shown in **Fig. 3A**, overexpressing dominant-negative mutants of TLR2, MyD88 or TRAF6 or TRAF7, a newly identified member of TRAF family, inhibited NF-κB activation by NTHi in human middle ear HMEEC-1 cells. Similar results were also observed in human airway epithelial A549 cells and human primary bronchial epithelial NHBE cells (Data not shown). Moreover, overexpressing dominant-negative mutants of MyD88, TRAF6 or TRAF7 also inhibited NTHi-induced expression of IL-1β and IL-8 in HMEEC-1 cells (**Fig. 3B**). Consistent with these *in vitro* findings, *Tlr2^−/−^* mice showed significant reduction in both IL-1β and MIP-2 expression, when mice were intratracheally inoculated with NTHi (**Fig. 3C**). Similar result was observed in *MyD88^+/−^* mice (**Fig. 3D**). Moreover, histological analysis of lungs of NTHi-inoculated mice revealed markedly reduced inflammatory response in *Tlr2^−/−^* mice compared with WT mice (**Fig. 3E**). Thus, it is evident that TLR2-dependent MyD88-TRAF6/7 signaling pathway plays a critical role in mediating NTHi-induced NF-κB-dependent inflammatory response *in vitro* and *in vivo*.

### CYLD acts as a negative regulator for NTHi-induced NF-κB activation via negative cross-talk with TRAF6/7

**Figure 4:**
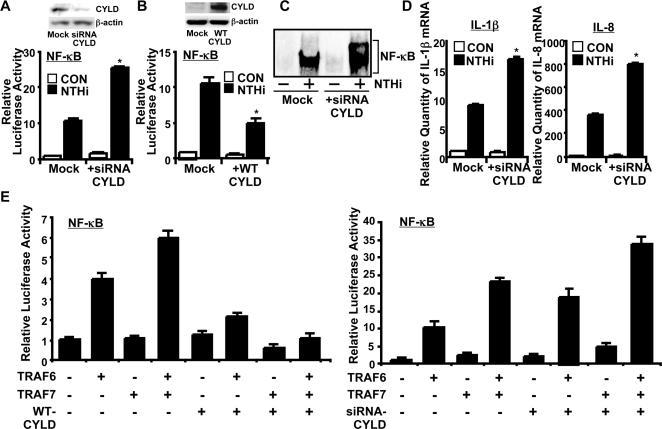
CYLD acts as a negative regulator for NTHi-induced NF-κB activation via negative cross-talk with TRAF6/7. A. CYLD knockdown by siRNA-CYLD markedly reduced expression of CYLD and enhanced NTHi-induced NF-κB-dependent promoter activity in HMEEC cells. B, overexpression of WT-CYLD inhibited NTHi-induced NF-κB-dependent promoter activity in HMEEC cells. C, NTHi-induced DNA binding activity of NF-κB was enhanced by CYLD knockdown, as assessed by EMSA. D, CYLD knockdown by siRNA-CYLD enhanced NTHi-induced IL-1β and IL-8 expression at mRNA level in HMEEC-1 cells. E, TRAF7 synergistically enhanced TRAF6-induced NF-κB-dependent promoter activity. Overexpression of WT-CYLD inhibited TRAF6- and TRAF7-induced NF-κB-dependent promoter activity, whereas CYLD knockdown by siRNA-CYLD enhanced TRAF6- and TRAF7-induced NF-κB-dependent promoter activity in lung epithelial A549 cells. *p<0.05, compared with NTHi-inoculated in Mock in A, B and D. Values are means ± S.D. (n = 3).

Having demonstrated the critical role of TLR2-dependent MyD88-TRAF6/7 signaling pathway in NTHi-induced NF-κB-dependent inflammatory response, we next sought to determine if CYLD negatively regulates NTHi-induced NF-κB activation by targeting TRAF6/7. We initially evaluated the role of CYLD in activation of NF-κB and the resultant cytokine induction by NTHi by CYLD knockdown in human middle ear epithelial HMEEC-1 cells. We first confirmed the efficiency of CYLD-specific siRNA (siRNA-CYLD) in reducing CYLD expression. As expected, endogenous CYLD protein was markedly reduced by siRNA (**Fig. 4A** **, upper panel**). CYLD knockdown by using siRNA-CYLD enhanced activation of NF-κB by NTHi, whereas overexpression of WT-CYLD inhibited it (**Fig. 4A** **, lower panel &** **Fig. 4B**). Moreover, CYLD knockdown enhanced NTHi-induced DNA binding activity of NF-κB (**Fig. 4C**). Consistent with these results, siRNA-CYLD also enhanced the induction of IL-1β and IL-8 expression by NTHi in middle ear epithelial HMEEC-1 cells (**Fig. 4D**). Similar results were also observed in human airway epithelial A549 cells and primary airway epithelial NHBE cells (Data not shown). Our data thus suggest that CYLD indeed acts as a negative regulator for NTHi-induced NF-κB-dependent inflammatory response in middle ear and respiratory epithelial cells.

We next sought to determine how CYLD inhibits NTHi-induced NF-κB activation. We previously showed that TRAF7 cooperates with TRAF6 in mediating NF-κB activation. Since we have shown that TRAF6 and 7 are involved in NTHi-induced NF-κB activation, it is likely that CYLD may inhibit NTHi-induced NF-κB activation by targeting TRAF6 and 7. We first tested our hypothesis by evaluating if CYLD knockdown or overexpressing WT CYLD alters NF-κB activation induced by expressing WT TRAF6 and 7. As shown in **Fig. 4E**, TRAF7 synergistically enhanced TRAF6-induced NF-κB activation, and overexpression of WT-CYLD inhibited TRAF6 and 7-induced NF-κB activation, whereas CYLD knockdown by siRNA-CYLD enhanced NF-κB activation induced by wt-TRAF6 and 7. Our data thus suggests that CYLD inhibits NF-κB by targeting TRAF6 and 7.

### CYLD inhibits TRAF6 and TRAF7 in a deubiquitination-dependent manner

**Figure 5:**
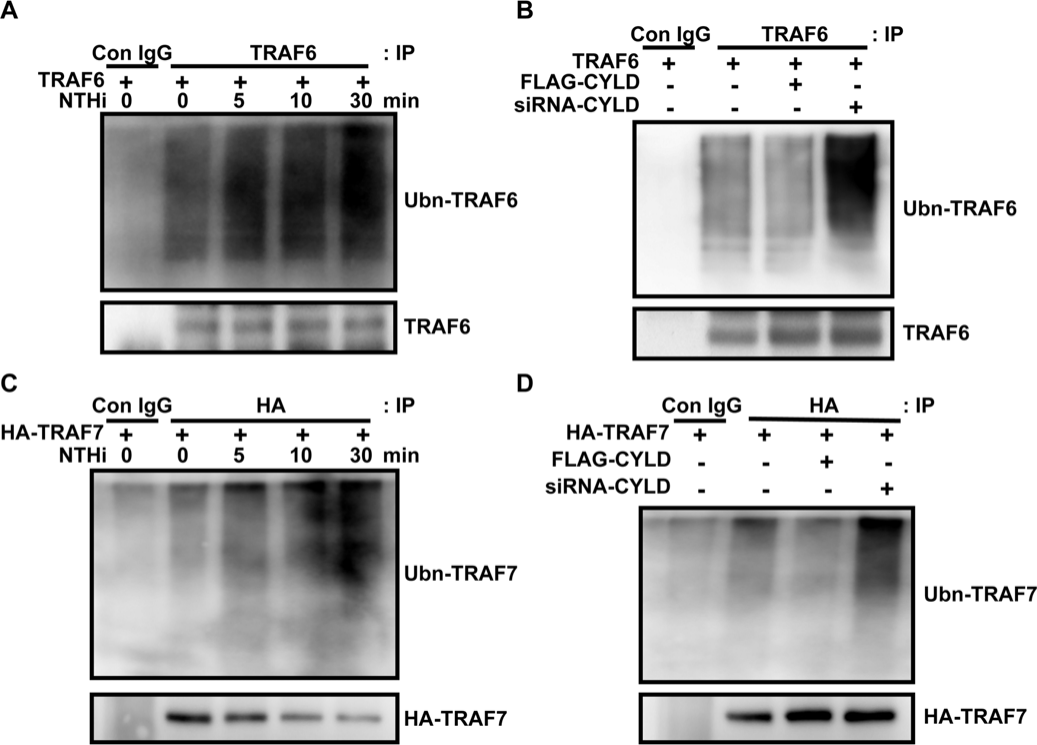
CYLD inhibits TRAF6 and TRAF7 in a deubiquitination-dependent manner. A, NTHi induced ubiquitination of TRAF6. B, co-expressing WT-CYLD inhibited, whereas CYLD knockdown by siRNA-CYLD enhanced the ubiquitination of TRAF6 in HeLa cells. C, NTHi induced ubiquitination of TRAF7. D, co-expressing WT-CYLD inhibited, whereas CYLD knockdown by siRNA-CYLD enhanced the ubiquitination of TRAF7 in HeLa cells.

Because CYLD is known as a deubiquitinating enzyme (DUB) and polyubiquitination plays an important role in TRAFs activation [16]–[18], [20], we next determined if CYLD deubiquitinates TRAF6 and 7. We first examined whether NTHi induces polyubiquitination of TRAF6 and TRAF7. As shown in the **Fig. 5A & C**, NTHi induced polyubiquitination of TRAF6 and TRAF7 in a time-dependent manner, respectively. Moreover, co-expression of WT-CYLD caused a reduction in TRAF6 and TRAF7 ubiquitination, and CYLD knockdown by siRNA-CYLD markedly increased levels of ubiquitinated TRAF6 and TRAF7 in HeLa cells (**Fig. 5B & D**). Together, we conclude that CYLD inhibits NTHi-induced NF-κB-dependent inflammatory response by deubiquitinating TRAF6 and TRAF7.

## Discussion

**Figure 6:**
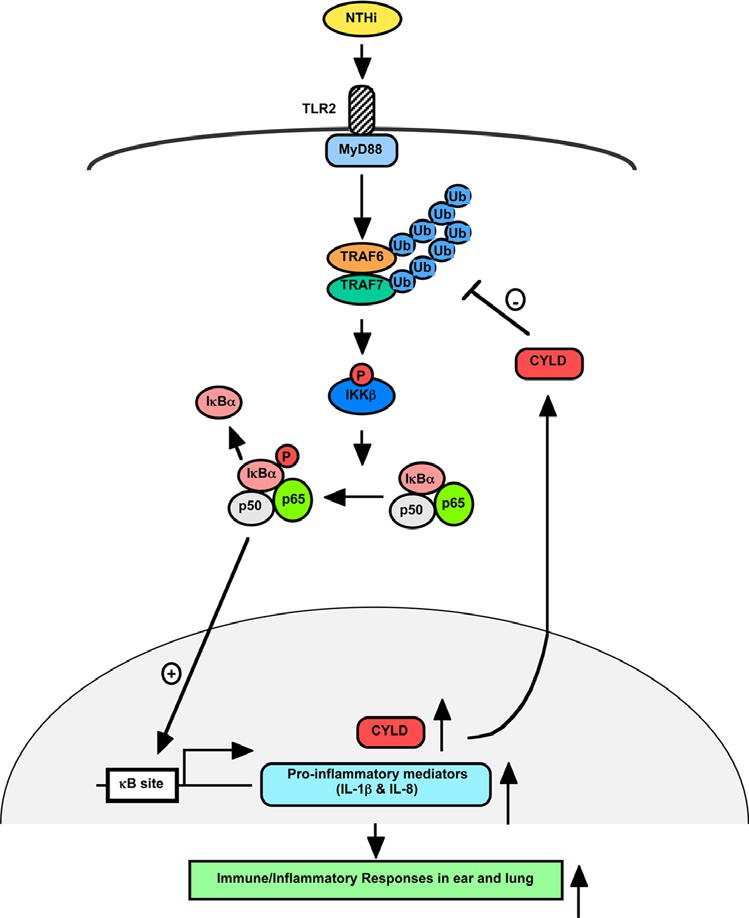
Schematic representation of the negative regulation of NTHi-induced inflammatory response by CYLD.

Negative feedback regulation plays a critical role in preventing overactive and detrimental inflammatory response in a variety of human diseases including infectious diseases [31]. To fully understand the molecular mechanisms underlying the negative regulation of inflammatory response in NTHi infections including otitis media and chronic obstructive pulmonary diseases, it is essential to identify the key receptor-mediated signaling adaptors. We previously showed that TLR2 is required for NTHi-induced NF-κB-dependent inflammatory response [6], [9], [10]. However, the key signaling adaptors that link TLR2 with NF-κB still remain unknown. In the present study, by overexpressing dominant-negative mutants of TLR2, MyD88 and TRAF6/7 and by using *Tlr2^−/−^* and *MyD88^+/−^* mice, we demonstrated that NTHi induced NF-κB-dependent inflammatory response through TLR2-dependent MyD88-TRAF6/7 signaling pathway (**Fig. 6**). This finding is of particular interest because disrupting the signaling-mediated by TRAFs, the bottleneck of the receptor-mediated signaling, should efficiently block NTHi-induced TLR2-dependent NF-κB activation and the subsequent inflammatory response. Thus, the TRAF6/7 complex appears to be an attractive target for therapeutic intervention [32]–[36]. Indeed, over the past decade significant effort has been put into developing therapeutic strategies to shut down TRAF6 signaling for reducing the mortality associated with septic shock [37]. Since TRAF6/7 complex is essential for transducing NTHi-initiated signal from surface TLR to the NF-κB in the nucleus, it would be thus an ideal target for negative feedback regulation in the pathogenesis of bacteria infection.

The deubiquitinating enzyme CYLD was initially identified as a tumor suppressor because loss of which causes a benign human tumor called cylindromatosis [13]–[15]. *In vitro* studies indicate that CYLD inhibits TNF-α-induced NF-κB activation by deubiquitinating TRAF2 [16]–[18]. Ubiquitination is previously known for its role in targeting proteins for degradation by the proteasome, but evidence of the nonproteolytic functions of ubiquitin is also rapidly accumulating. Initial evidence for the regulatory, rather than proteolytic, function of ubiquitin is provided by study of the TRAF proteins, which function as ubiquitin ligases to synthesize lysine 63 (K63)-linked polyubiquitin chains to mediate activation of downstream kinases through a proteasome-independent mechanism [38]. Despite the role of CYLD in inhibiting TRAF2 by deubiquitinating it, its role in deubiquitinating other TRAF family members remains unclear. The role of CYLD in regulating NTHi-induced NF-κB activation also remains unknown. Moreover, despite its known role in regulating T-cell development and tumor cell proliferation *in vivo* [39], [40], its role in regulating inflammation in bacterial infections *in vivo* is still unclear. In the present study, we have shown that, by performing the inflammatory phenotype analysis of *Cyld^−/−^* mice, CYLD negatively regulates NTHi-induced inflammatory response in the middle ear and lung of *Cyld^−/−^* mice (**Fig. 1**), and this negative regulation results in enhanced pathological changes in the middle ear and lung as well as increased expression of pro-inflammatory mediators *in vivo*. Thus our data provide direct *in vivo* evidence for the negative regulation of CYLD in NTHi-induced inflammation via a deubiquitination-dependent inhibition of TRAF6/7 in the middle ear and lungs of mice.

In the present study, it is clear that CYLD acts as a critical negative regulator for NTHi-induced inflammation in the middle ear and lung. However, the expression of CYLD in the middle ear and lung is relatively low under physiological condition. Moreover, *Cyld^−/−^* mice exhibit no overt abnormalities under normal conditions [21], [40]. Moreover it was recently reported that *Cyld^−/−^* mice are more susceptible to chemical-induced skin-tumorigenesis even though they appear normal under physiological condition. These interesting findings thus led us to hypothesize that CYLD, although expressed at relatively low level under physiological conditions, is up-regulated by bacteria during middle ear and lung infection, which in turn leads to the attenuation of NTHi-induced inflammation. In view of CYLD in negative regulation of bacteria-induced TLR2-dependent inflammatory response, it is interesting to note that CYLD expression itself is also regulated by bacteria-induced TLR activation [20], [23]. CYLD is induced rapidly after activation of TLR2 by various bacterial pathogens including PGN, MALP-2, Pam3CSK4, and NTHi. Our finding reveals novel molecular mechanisms by which bacterial pathogens including NTHi induce CYLD expression, which in turn attenuates bacteria-induced inflammatory response. Given that prolonged CYLD expression is anticipated to suppress adequate host response to the invading pathogens, which is necessary for the adequate host defense, it is also of particular interest to investigate how CYLD expression is negatively regulated. This study should provide novel insights into how inflammation is tightly regulated and may lead to the development of novel therapeutic strategies for modulating inflammation.
